# c-Jun N-terminal kinase in synergistic neurite outgrowth in PC12 cells mediated through P90RSK

**DOI:** 10.1186/1471-2202-14-153

**Published:** 2013-12-12

**Authors:** Kok Huei Seow, Lihan Zhou, Gregory Stephanopoulos, Heng-Phon Too

**Affiliations:** 1 Chemical & Pharmaceutical Engineering, Singapore-MIT Alliance, 4 Engineering Drive 3, Singapore, Singapore; 2 Department of Biochemistry, National University of Singapore, 14 Medical Drive, Singapore, Singapore; 3 Department of Chemical Engineering, Massachusetts Institute of Technology, 77 Massachusetts Avenue, Cambridge, USA

**Keywords:** PC12, Synergistic, NGF, FGFb, EGF, PACAP, Neurite outgrowth, JNK, P90RSK

## Abstract

**Background:**

Synergistic multi-ligand treatments that can induce neuronal differentiation offer valuable strategies to regulate and modulate neurite outgrowth. Whereas the signaling pathways mediating single ligand-induced neurite outgrowth, such as Akt, extracellular signal-regulated kinase (Erk), c-Jun N-terminal kinase (JNK), and p38 mitogen-activated protein kinase (P38), have been extensively studied, the mechanisms underlying multi-ligand synergistic neurite outgrowth are poorly understood. In an attempt to gain insight into synergistic neurite outgrowth, PC12 cells were treated with one of three combinations: pituitary adenylate cyclase-activating peptide (PACAP) with epidermal growth factor (EP), basic fibroblast growth factor (FP), or nerve growth factor (NP) and then challenged with the appropriate kinase inhibitors to assess the signaling pathways involved in the process.

**Results:**

Response surface analyses indicated that synergistic neurite outgrowth was regulated by distinct pathways in these systems. Synergistic increases in the phosphorylation of Erk and JNK, but not Akt or P38, were observed with the three growth factor-PACAP combinations. Unexpectedly, we identified a synergistic increase in JNK phosphorylation, which was involved in neurite outgrowth in the NP and FP, but not EP, systems. Inhibition of JNK using the SP600125 inhibitor reduced phosphorylation of 90 kDa ribosomal S6 kinase (P90RSK) in the NP and FP, but not EP, systems. This suggested the involvement of P90RSK in mediating the differential effects of JNK in synergistic neurite outgrowth.

**Conclusions:**

Taken together, these findings reveal the involvement of distinct signaling pathways in regulating neurite outgrowth in response to different synergistic growth factor-PACAP treatments. Our findings demonstrate a hitherto unrecognized mechanism of JNK-P90RSK in mediating synergistic neurite outgrowth induced by the co-treatment of growth factors and PACAP.

## Background

The effects of synergistic activity have been gaining attention in the treatment of diseases such as cancer and AIDS [1,2]. Drug or ligand synergy is defined as the joint action of two or more agents for which the result is greater than the sum of the actions of the individual parts. Synergistic therapeutic strategies therefore have the potential to accelerate the response to treatment, achieve higher efficacy, and potentially reduce the side effects associated with single treatment approaches [3-5]. Indeed, several studies have demonstrated the benefits of the co-administration of neurotrophic factors [6] and the combinatorial treatment of nerve growth factor (NGF) with glial cell-derived neurotrophic factor (GDNF) [7] or insulin-like growth factor (IGF)-1 [8] in promoting synergistic axonal or neurite elongation. Axonal and dendritic outgrowth from the neuronal cell body is a key cellular aspect of neuronal differentiation that is critically important not just in development, but also in recovery from injuries and neurodegenerative diseases [9]. Following nerve injury, the rate of nerve regeneration is critical, as a full functional recovery can be impeded by delayed regeneration [10]. Thus, accelerating or facilitating neurite outgrowth through a synergistic approach may provide a valuable treatment strategy for patients with nerve injury. However, the mechanisms underlying such synergistic action are currently not well understood.

Rat pheochromocytoma PC12 cells have been widely used as a model to study neuronal differentiation. Upon treatment with ligands such as the NGF [11], basic-fibroblast growth factor (FGFb or FGF-2) [12], and pituitary adenylate cyclase-activating peptide (PACAP) [13], PC12 cells differentiate into sympathetic neuron-like cells characterized by long-term and stable neurite outgrowth. These ligands regulate neurite outgrowth through pathways such as the extracellular signal-regulated kinase 1/2 (Erk) [14,15], p38 mitogen-activated protein kinase (P38) [16], c-Jun N-terminal kinase (JNK) [15,17], and the phosphatidylinositol 3-kinase (PI3K) [18]. In comparison, epidermal growth factor (EGF) promotes proliferation instead of differentiation in these cells [19,20]. Studies have attributed this difference in cell fate to the kinetics of Erk activation, where transient or sustained Erk activation leads to proliferation or differentiation, respectively [14,21]. Although EGF alone does not induce neurite outgrowth in PC12 cells, it has been found to synergize with cyclic adenosine monophosphate (cAMP)-elevating agents such as PACAP and forskolin, thereby enhancing neurite outgrowth [22,23]. Consistent with the knowledge that Erk is important in regulating differentiation, enhanced Erk activity has also been observed in the synergy model [23]. Similarly, cAMP-elevating agents have also been found to synergize with FGFb [24] and NGF [25,26] to enhance neurite outgrowth, where both P38 and Erk have been found to regulate neurite outgrowth induced by NGF-cAMP [26,27]. Whereas NGF, FGFb and EGF can all cooperate with cAMP-elevating agents to enhance neurite outgrowth, an interesting question is whether these three systems activate a common set of signaling pathways to mediate such synergy.

In this study, we investigated the activation and involvement of various signaling pathways in synergistic neurite outgrowth using three combinations of ligands: NGF-PACAP (NP), FGFb-PACAP (FP) and EGF-PACAP (EP). As expected, all three systems showed a synergistic phosphorylation of Erk concomitant with neurite outgrowth. Interestingly, JNK, but not Akt or P38, was also synergistically activated in all three systems. Unexpectedly, inhibition of JNK blocked neurite outgrowth in the NP and FP, but not EP, systems. This differential involvement of JNK was found to be dependent on the regulation of P90RSK activity. Thus, a JNK-P90RSK link was identified as a hitherto unrecognized mechanism mediating the synergistic effect in neurite outgrowth. Our results therefore demonstrate the involvement of distinct signaling pathways in regulating neurite outgrowth in response to different synergistic growth factor-PACAP stimulation.

## Methods

### Materials

Mouse recombinant NGF was purchased from Peprotech (Rocky Hill, NJ). Mouse recombinant EGF was purchased from Shenandoah Biotechnology (Warwick, PA). PACAP was purchased from American Peptide Company (Sunnyvale, CA). MEK inhibitor U0126, JNK inhibitor SP600125, PI3K inhibitor LY294002, and P38 inhibitor SB203580 were purchased from LC Laboratories (Woburn, MA). P90RSK inhibitor BRD7389 was purchased from Santa Cruz Biotechnology (Santa Cruz, CA). Primary antibodies against phospho-specific Erk1/2 (Thr202/Tyr204) (pErk), pan-Erk1/2, phospho-specific JNK (Thr183/Tyr185) (pJNK), pan-JNK, phospho-specific P38 (Thr180/Tyr182) (pP38), phospho-specific Akt (Ser473) (pAkt), phospho-specific P90RSK (Ser380) (pP90RSK), and pan-RSK were purchased from Cell Signaling Technologies (Danver, MA). An antibody against phospho-specific c-Jun (Ser73) (pc-Jun) was purchased from Abnova (Taipei, Taiwan). Human recombinant FGFb and an antibody against actin were purchased from EMD Millipore (Billerica, MA). Horseradish peroxidase-conjugated secondary antibodies, Imperial Protein Stain and Hoechst were purchased from Thermo Scientific (Wilmington, DE).

### Cell culture

Rat pheochromocytoma PC12 cells (American Type Culture Collection, Manassas, VA) were cultured in Dulbecco’s minimum essential medium (DMEM) supplemented with 10% heat inactivated fetal bovine serum (FBS, Sigma-Aldrich, St. Louis, MO) and 5% Horse Serum (HS, Hyclone, Thermo Scientific). Cells were cultured with 100U/ml penicillin and 100 mg/ml streptomycin, and maintained in a humidified incubator with 5% CO_2_ at 37°C.

### Western blot analyses

PC12 cells were seeded into the wells of 6-well plates pre-coated with poly-D-lysine at a density of 500,000 cells/well and cultured in growth medium for 48 hours. Following this, cells were incubated in serum-depleted medium (1% FBS, 0.5% HS) for an additional 16 hours. Cells were then simulated with individual or combinations of NGF, FGFb, EGF, and PACAP. For treatments with inhibitors, the cells were pre-incubated for 1 hour with the respective inhibitors prior to stimulations with the ligands. Cells were harvested within 1 hour after ligand stimulation. Treated cells were washed once with PBS and subsequently lysed in 2% sodium dodecyl sulfate (SDS). Protein concentrations in the total cell lysates were quantified using the microBCA assay (Pierce Biotechnology, Rockford, IL). The protein samples were then separated by SDS-polyacrylamide gel electrophoresis (SDS-PAGE), transferred onto nitrocellulose membranes, blocked with 5% milk and probed with antibodies against phosphorylated (p)Erk (1:5000 dilution), pJNK (1:1000 dilution), pAkt (1:1000 dilution), pP38 (1:1000 dilution), pP90RSK (1:1000 dilution), pc-Jun (1:1000 dilution), total Erk (1:5000 dilution), total JNK (1:1000 dilution), total P90RSK (1:1000 dilution), and actin (1:10,000 dilution). Blots were stripped with Restore Western Stripping Buffer (Pierce Biotechnology) and re-probed for different proteins. The protein bands were developed with Immobilon Western Chemiluminescent HRP Substrate (Millipore) on a ChemiDoc XRS system (Biorad, Hercules, CA). The band intensities were quantified using Quantity One 1-D Analysis software (Biorad). To enable comparisons of signals across different blots, lysates from NGF-PACAP–treated PC12 cells were used to generate a standard curve for each blot.

### Measurement of neurite outgrowth

PC12 cells were seeded into the wells of 12-well plates at a density of 25,000 cells/well, and cultured as described for western blotting. After treatment with the respective ligands for 48 hours, the cells were fixed with 4% paraformaldehyde for 20 minutes and permeabilized with ice-cold methanol for 15 minutes. The cell bodies were then stained with Imperial Protein Stain for 15 minutes and the nuclei with Hoechst stain for 5 minutes. The images of the cells were then captured using a Zeiss inverted fluorescent microscope (Zeiss Oberkochen, Germany). The length of the neurites was quantified using HCA-Vision software (CSIRO, North Ryde, NSW, Australia). The neurite quantification procedure, which involved neuron body detection, neurite detection, and neurite analysis, was performed as previously described [28]. The neurite length obtained under control conditions (i.e., in the absence of NGF and PACAP) was subtracted from each treatment condition. Thereafter, the neurite length for each condition was normalized against that obtained for cells grown under 50 ng/ml of NGF, assigned an arbitrary value of 1.

### Statistical analyses

Statistical significance was determined using the Student’s *t-*test and the respective results are displayed as the mean ± standard deviation (S.D.). All experiments and measurements were replicated at least three times.

## Results

### Response surface analyses suggests that synergistic neurite outgrowth is regulated by discrete mechanisms in different systems

**Figure 1:**
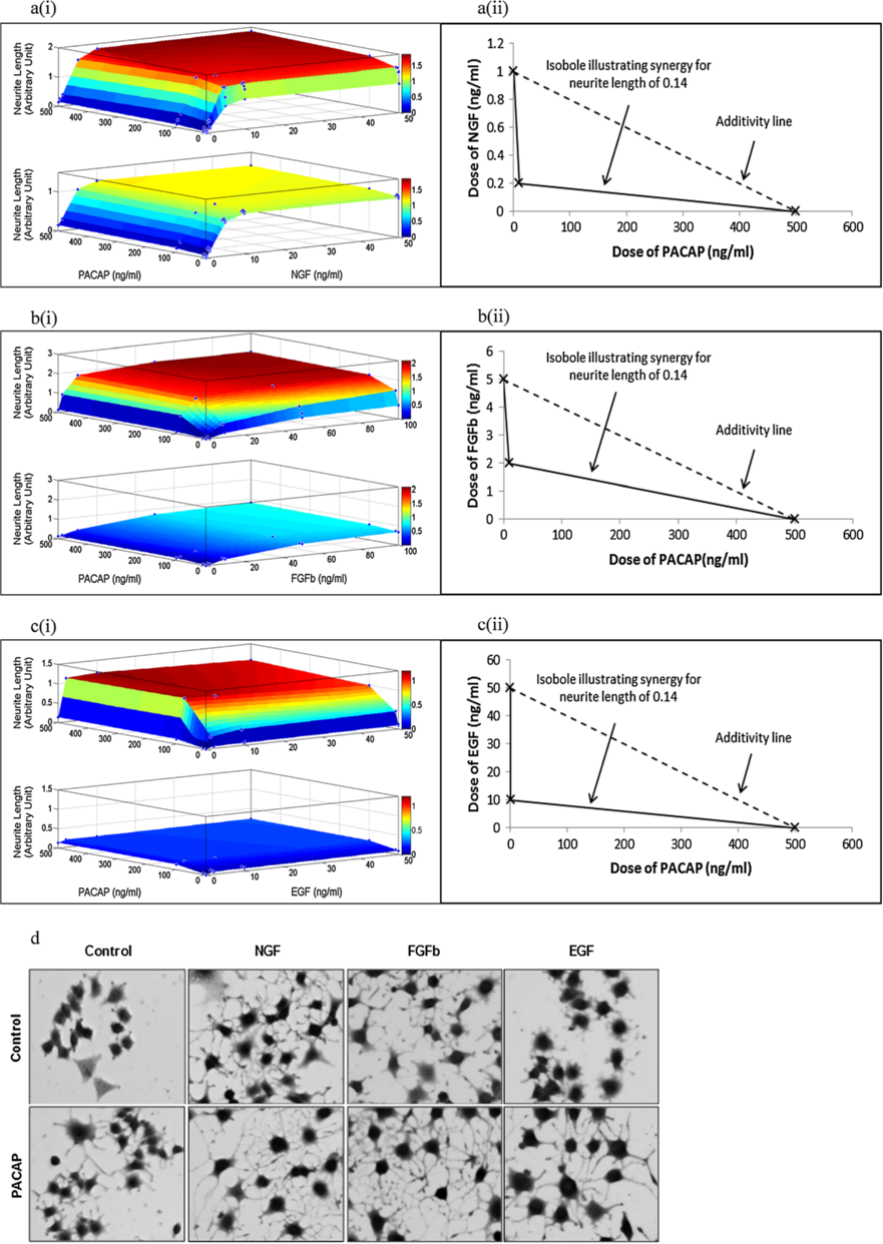
**Synergistic neurite outgrowth induced by combinatorial growth factor-PACAP treatments. (a(i))**, **(b(i))**, **(c(i))** Response surface plots for the NGF (0-50 ng/ml)-PACAP (0-500 ng/ml) (NP), FGFb (0-100 ng/ml)-PACAP (0-500 ng/ml) (FP), and EGF (0-50 ng/ml)-PACAP (0-500 ng/ml) (EP) systems, respectively. Top panel: Experimentally obtained results of the growth factor-PACAP combinatorial treatment; Bottom panel: Additive effect calculated through the summation of the individual effects of the growth factors and PACAP. The x, y, and z axes denote concentrations of PACAP (ng/ml), concentrations of growth factors (ng/ml), and neurite length, respectively. **(a(ii))**, **(b(ii))**, **(c(ii))** Isobologram plots illustrating the concentrations of growth factor and PACAP necessary to obtain a neurite length of 0.14 for the NP, FP, and EP systems, respectively. **(d)** Representative images of cells treated with each growth factor (50 ng/ml) with and without PACAP (100 ng/ml).

NGF [25,26], FGFb [24] and EGF [22,23] are known to synergize with cAMP-elevating agents to enhance neurite outgrowth. NGF or FGFb cause considerably longer neurite outgrowth than EGF or PACAP (Figure 1). To better visualize the synergistic action between growth factors and PACAP on neurite length, we used a response surface model (RSM) [29] and examined the effect of NGF-PACAP (NP), FGFb-PACAP (FP) and EGF-PACAP (EP) treatments in these cells. The cells were treated with the ligands singly and in combination. In these plots, the neurite length obtained after 48 hours of combinatorial treatment was compared to that obtained by a summation of neurite length induced by the individual ligands (additive effect). Surface plots of the three systems—NP (Figure 1a(i)), FP (Figure 1b(i)), and EP (Figure 1c(i))—clearly indicated that combinatorial treatments resulted in longer neurites than the additive effects of single ligand exposure, indicating synergism. These plots also showed that synergism (as indicated by the plateau regions) occurred over a wide range of doses of growth factors and PACAP. To further illustrate that synergistic neurite outgrowth can occur even with low doses of PACAP, an isobologram [30] was plotted for each of the three systems (Figure 1a(ii), 1b(ii), &1c(ii)). Significantly higher concentrations of PACAP were required in the absence of any growth factors to obtain similar neurite lengths. In addition, in the NP and FP systems, the saturating neurite length for the combinatorial treatment was about twice that of the additive effect, whereas a difference of about 4-fold was observed for the EP system. This indicates a higher degree of synergism in the EP system, and suggests that synergistic neurite outgrowth in the EP system may differ mechanistically from those of the NP and FP systems. Representative images of the neurite outgrowth in each system are shown in Figure 1d.

### Synergistic phosphorylation of Erk & JNK upon combinatorial growth factor-PACAP treatment

We hypothesized that there was likely to be synergistic activation of the various kinases that regulate synergistic neurite outgrowth. To examine the pathways involved in regulating synergistic neurite outgrowth in these systems, we conducted a time-course to determine changes in the phosphorylation levels of four kinases—Akt, Erk, JNK, and P38—upon NGF, PACAP, and NP treatments. The kinases were activated throughout the entire 1 hour time-course (Additional file 1: Figure S1). Thus, for convenience, subsequent analyses were performed only at 20 and 60 minutes time-points.

**Figure 2:**
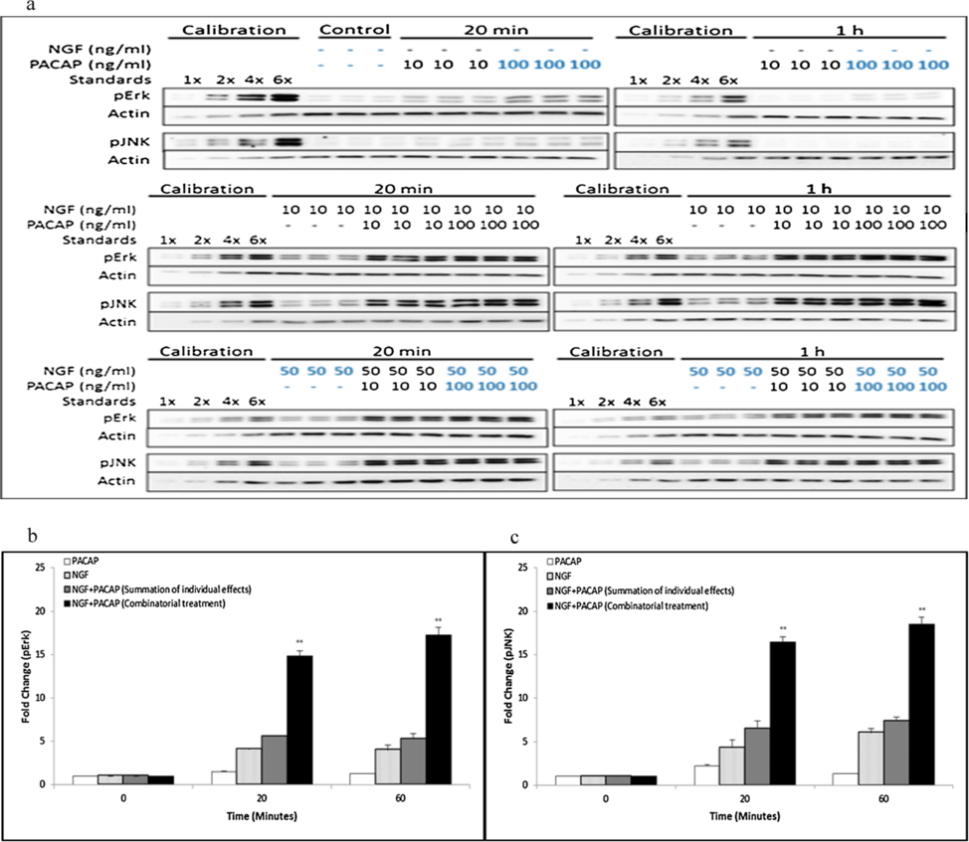
**Synergistic and sustained phosphorylation of Erk and JNK upon combinatorial NGF and PACAP treatment. (a)** Time-course of Erk and JNK phosphorylation at 20 and 60 minutes following NGF (0-50 ng/ml)-PACAP (0-100 ng/ml) (NP) treatment. Phosphorylation levels of the proteins were analyzed by western blotting, and normalized to the levels of actin. Fold-changes in **(b)** pErk and **(c)** pJNK were quantified by densitometry. Data for 50 ng/ml NGF and 100 ng/ml PACAP (highlighted in blue) were plotted and analyzed for synergism. Significant differences between combinatorial experimental treatment of NGF-PACAP and summation of their individual effects were calculated using the paired Student’s *t*-test. A value of *p* < 0.05 was considered significant (**p < 0.01). Graphs showing synergistic Erk and JNK phosphorylation at the other ligand concentrations were plotted in Additional file 3: Figure S3a & S3b. Western blot data and quantified results for pP38 and pAkt were plotted in Additional file 2: Figure S2a-S2c.

After treating the cells with multiple doses of NGF and PACAP, the phosphorylation levels of Erk, JNK (Figure 2a), P38 and Akt (Additional file 2: Figure S2a) were quantified and analyzed for synergism. Single ligand treatment with NGF but not PACAP induced sustained Erk phosphorylation. To analyze for synergistic activation of Erk, effects upon combinatorial treatments of NP was compared to the additive effect of the individual ligands. In the presence of both ligands, Erk phosphorylation was higher than the additive effects of NGF and PACAP separately (Figure 2b, & Additional file 3: Figure S3a). This is in congruence with the finding that NGF and NP treatment but not PACAP induced extensive neurite outgrowth, and is consistent with the idea that sustained Erk phosphorylation is involved in neurite outgrowth [21,27,31].

Similarly, sustained activation of JNK by NGF was observed (Figure 2c). In addition, we made the novel discovery that JNK was also synergistically phosphorylated upon combinatorial NP treatment (Figure 2c, & Additional file 3: Figure S3b) and it was sustained for up to 1 hour post-stimulation. On the contrary, using the same analyses, synergistic phosphorylation of P38 (Additional file 2: Figure S2a, & S2b) and Akt (Additional file 2: Figure S2a, & S2c) were not observed in the NP system.

**Figure 3:**
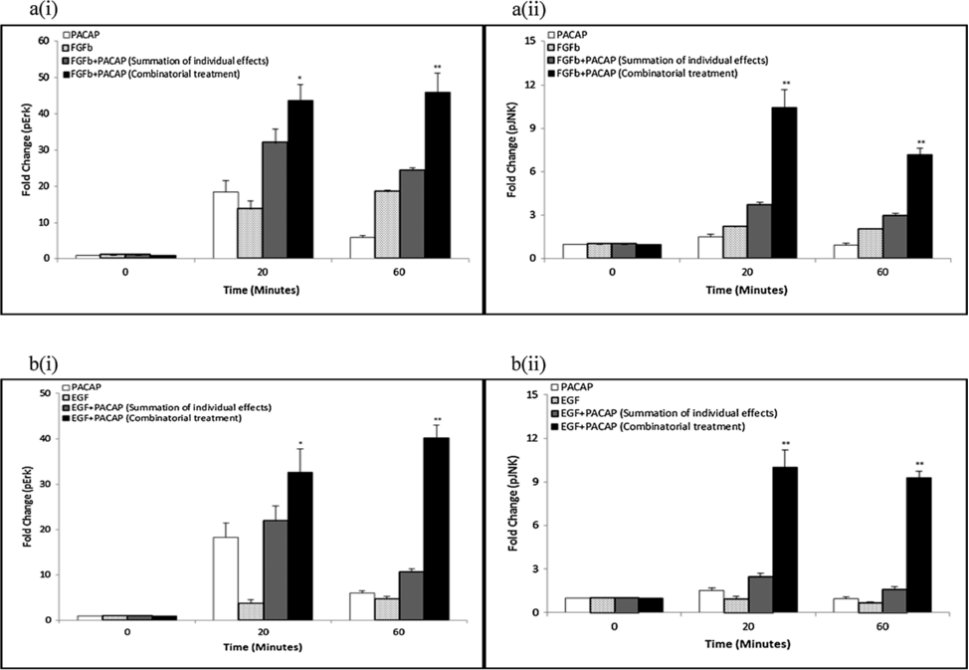
**Synergistic and sustained phosphorylation of Erk and JNK upon FP and EP treatments.** Time-course of quantified Erk and JNK phosphorylation at 20 and 60 minutes upon **(a)** FGFb (50 ng/ml)-PACAP (100 ng/ml) (FP) or **(b)** EGF (50 ng/ml)-PACAP (100 ng/ml) (EP) treatment. Fold-change in (i) pErk, and (ii) pJNK were quantified by densitometry and normalized to the levels of actin. Significant differences between combinatorial experimental treatment of growth factor-PACAP and the summation of their individual effects were calculated using the paired Student’s *t*-test. A value of *p* < 0.05 was considered significant (**p < 0.01; *p < 0.05).

Having found that Erk and JNK were synergistically phosphorylated in the NP system, we next investigated if these trends were also common to the FP and EP systems. Similar to the NP system, sustained and synergistic Erk (Figure 3a(i), & b(i)) and JNK (Figure 3a(ii), & b(ii)) phosphorylation were observed for the FP and EP treatments, respectively, within 1 hour of stimulation. Likewise, neither P38 (Additional file 4: Figure S4a(i), & S4b(i)) nor Akt (Additional file 4: Figure S4a(ii), & S4b(ii)) were synergistically phosphorylated in the FP and EP systems. Thus, these results indicate that specific kinases were synergistically phosphorylated by growth factor-PACAP co-treatment, suggestive of their roles in mediating synergistic neurite outgrowth.

The total protein levels of Erk, JNK, P38 and Akt upon treatment with single ligand or combinations of the growth factors and PACAP were unchanged across all conditions and time-points (Additional file 5: Figure S5).

### Erk is required for neurite outgrowth in all 3 systems whereas JNK is required only for the NP and FP, but not EP, systems

**Figure 4:**
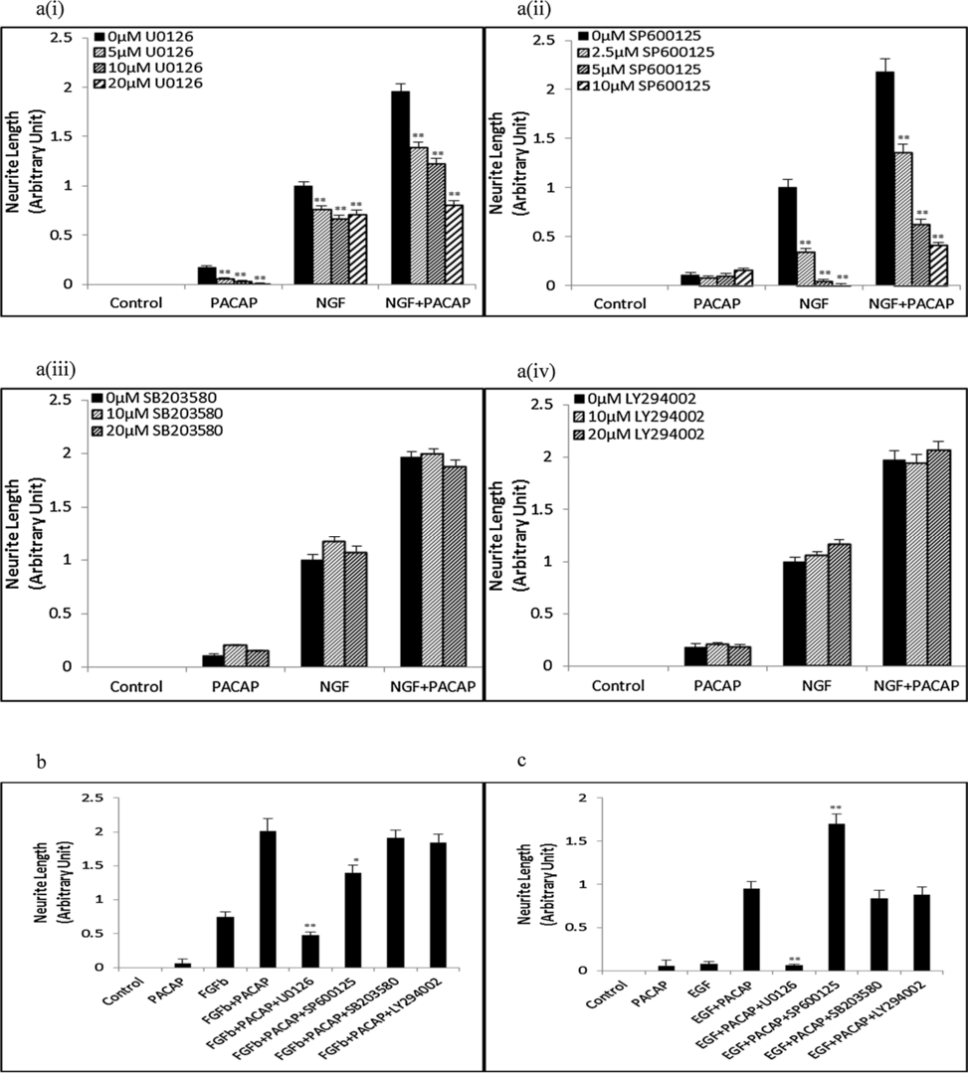
**Erk is required for neurite outgrowth in all 3 systems whereas JNK is required only for the NP and FP, but not EP, systems.** Concentrations of growth factors and PACAP used were 50 ng/ml and 100 ng/ml, respectively. **(a)** Dose–response treatment of (i) MEK inhibitor (U0126), (ii) JNK inhibitor (SP600125), (iii) P38 inhibitor (SB203580), and (iv) PI3K inhibitor (LY294002) on neurite outgrowth in the NP system. **(b)**, **(c)** Effect of U0126 (20 μM), SP600125 (10 μM), SB203580 (20 μM), and LY294002 (20 μM) on neurite outgrowth in the FP, and EP systems, respectively. Significant differences between treatments with and without inhibitors were calculated using the paired Student’s *t*-test. A value of *p* < 0.05 was considered significant (**p < 0.01; *p < 0.05).

We next examined the role of these synergistically activated kinases in regulating neurite outgrowth using kinase inhibitors. As expected, treatment with the MEK inhibitor, U0126, inhibited neurite outgrowth in the NP system in a dose-dependent manner (Figure 4a(i), & Additional file 6: Figure S6). Similarly, inhibition of MEK also blocked neurite outgrowth in the FP and EP systems (Figure 4b, c, & Additional file 6: Figure S6), confirming the involvement of synergistic Erk phosphorylation in neurite outgrowth. Further supporting the involvement of synergistically phosphorylated kinases in regulating synergistic neurite outgrowth, the JNK inhibitor, SP600125, blocked neurite outgrowth in the NP (Figure 4a(ii), & Additional file 6: Figure S6) and FP systems (Figure 4b, & Additional file 6: Figure S6). Surprisingly, SP600125 at the same concentration (10 μM) failed to inhibit neurite outgrowth in the EP system, showing instead enhanced neurite outgrowth (Figure 4c, & Additional file 6: Figure S6). Higher concentrations of SP600125 were deemed to be cytotoxic (data not shown). Positive controls for the effects of U0126 and SP600125 are shown in Additional file 7: Figure S7a and S7b, respectively.

As expected, inhibition of the non-synergistically activated nodes, P38 and Akt, by SB203580, and LY294002, respectively, did not block neurite outgrowth in all three systems (Figure 4a(iii)-(iv), b, c, & Additional file 6: Figure S6). Likewise, cells treated with doses of the inhibitors at concentrations higher than 20 μM resulted in high levels of cytotoxicity (data not shown). The positive controls for SB203580 and LY294002 are shown in Additional file 7: Figure S7c and S7d, respectively.

**Figure 5:**
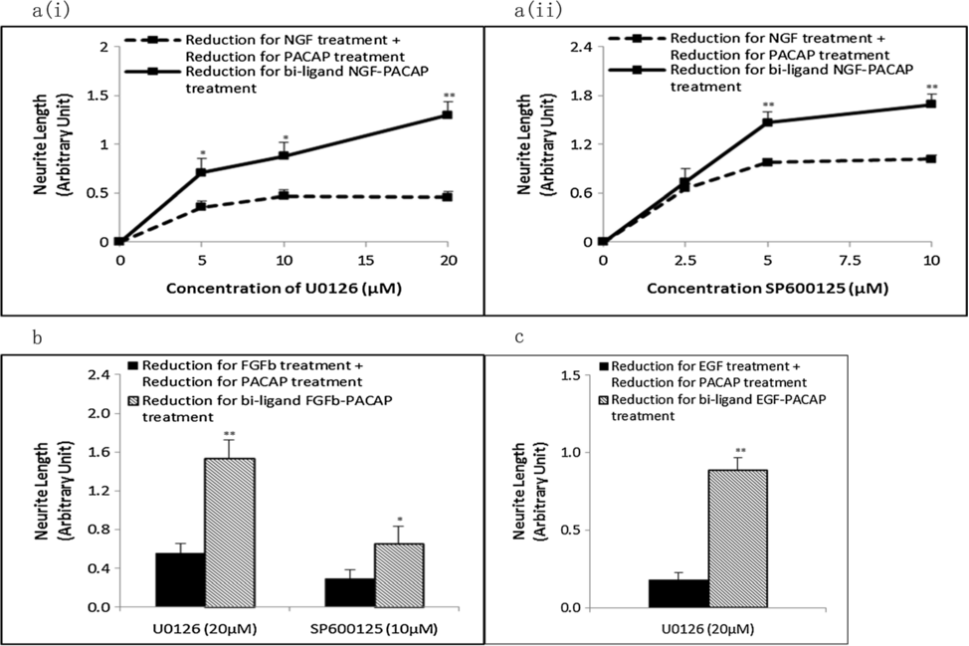
**Net inhibitor-induced reduction in neurite length is greater in the synergistic systems than in the additive effect of the single ligand treatments.** Net reduction in neurite outgrowth in the **(a)** NP system following treatment with various concentrations of (i) U0126 (MEK inhibitor) and (ii) SP600125 (JNK inhibitor). Reductions in neurite outgrowth in the **(b)** FP and **(c)** EP systems in the presence of specific kinase inhibitors. Significant differences between the effects of the combinatorial treatment of growth factor-PACAP (bi-ligand) versus the sum of the effects for each single ligand treatment were compared using the paired Student’s *t*-test. A value of *p* < 0.05 was considered significant (**p < 0.01; *p < 0.05).

Next, the reduction in neurite outgrowth, after treatment with inhibitors, for the NP treatment was compared to the sum of reduction of neurite outgrowth in the single ligand treatments. With U0126 (Figure 5a(i)) and SP600125 (Figure 5a(ii)), the reduction in neurite outgrowth in the NP treatment was greater than the sum of reduction for the single ligand treatments. Similarly, for the FP (Figure 5b) and EP (Figure 5c) systems, inhibition of the kinases required for neurite outgrowth also resulted in a greater reduction in neurite outgrowth in the combinatorial growth factor-PACAP treatments than the sum of reduction for the respective single ligand treatments. These results support the involvement of the various kinases in regulating synergistic neurite outgrowth in the respective synergistic systems.

Critically, these results also suggest that these systems utilize distinct pathways to regulate neurite outgrowth and that not all synergistically phosphorylated kinases are relevant to neurite outgrowth.

### P90RSK is a downstream target of both Erk & JNK in the NP & FP systems but is only downstream of Erk in the EP system

Having found that JNK was involved in neurite outgrowth in the NP and FP, but not EP, systems, we sought to identify the downstream targets that may be involved in mediating this differential requirement of JNK. Among the many downstream effectors of JNK, P90RSK has been recently shown to be involved in neurite outgrowth and PC12 cells differentiation [32-34]. Thus, we examined if P90RSK was synergistically phosphorylated and if it was involved in JNK-mediated neurite outgrowth.

**Figure 6:**
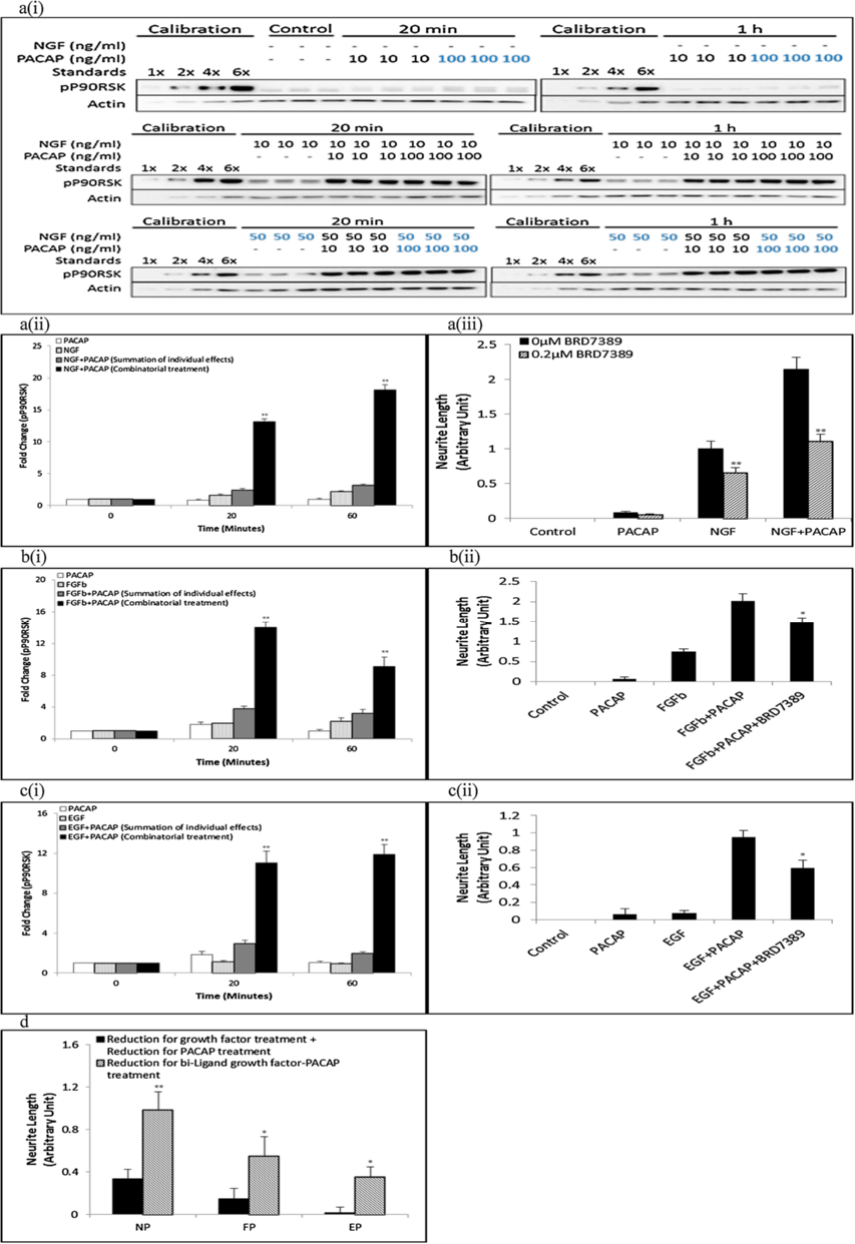
**P90RSK is synergistically phosphorylated and is involved in neurite outgrowth in all three systems. (a(i))** Time-course of P90RSK phosphorylation at 20 and 60 minutes following NGF (0-50 ng/ml)-PACAP (0-100 ng/ml) treatment. Phosphorylation levels of the proteins were analyzed by Western blotting, and normalized to the levels of Actin. The blots used were the same as those used for pJNK in Figure 2a. **(a(ii))**, **(b(i))**, **(c(i))** Time-course measurements of pP90RSK at 20 and 60 minutes following NGF (50 ng/ml)-PACAP (100 ng/ml) (NP), FGFb (50 ng/ml)-PACAP (100 ng/ml) (FP) or EGF (50 ng/ml)-PACAP (100 ng/ml) (EP) stimulations, respectively. The total protein levels of P90RSK upon treatment with single ligand or combinations of the growth factors and PACAP were unchanged across all conditions and time-points (Additional file 5: Figure S5). **(a(iii))**, **(b(ii))**, **(c(ii))** Effect of P90RSK inhibitor, BRD7389 (0.2 μM), on neurite outgrowth in the NP, FP, and EP systems, respectively. **(d)** Net reduction in neurite outgrowth between combinatorial ligand treatment (bi-ligand) versus the sum of neurite outgrowth reduction from treatment with each ligand separately in the presence of BRD7389 (0.2 μM). Significant differences between the effects of combinatorial experimental treatment of growth factor-PACAP and summation of their individual effects, and that between the effects of treatments with and without inhibitors were calculated using the paired Student’s *t*-test. A value of *p* < 0.05 was considered significant (**p < 0.01; *p < 0.05).

As expected, P90RSK was synergistically phosphorylated in the NP (Figure 6a(i)-a(ii), & Additional file 8: Figure S8a), FP (Figure 6b(i)) and EP (Figure 6c(i)) systems from 20 minutes to 1 hour after stimulation. In all three systems, neurite outgrowth was inhibited in the presence of the P90RSK inhibitor, BRD7389 [35,36] (Figure 6a(iii), b(ii), c(ii), & Additional file 8: Figure S8b). In these systems, greater reductions in neurite outgrowth were also achieved in the combinatorial growth factor-PACAP treatments than for the sum of the reduction in neurite outgrowth in the respective single ligand treatments (Figure 6d), supporting the involvement of P90RSK in regulating synergistic neurite outgrowth in all three systems.

**Figure 7:**
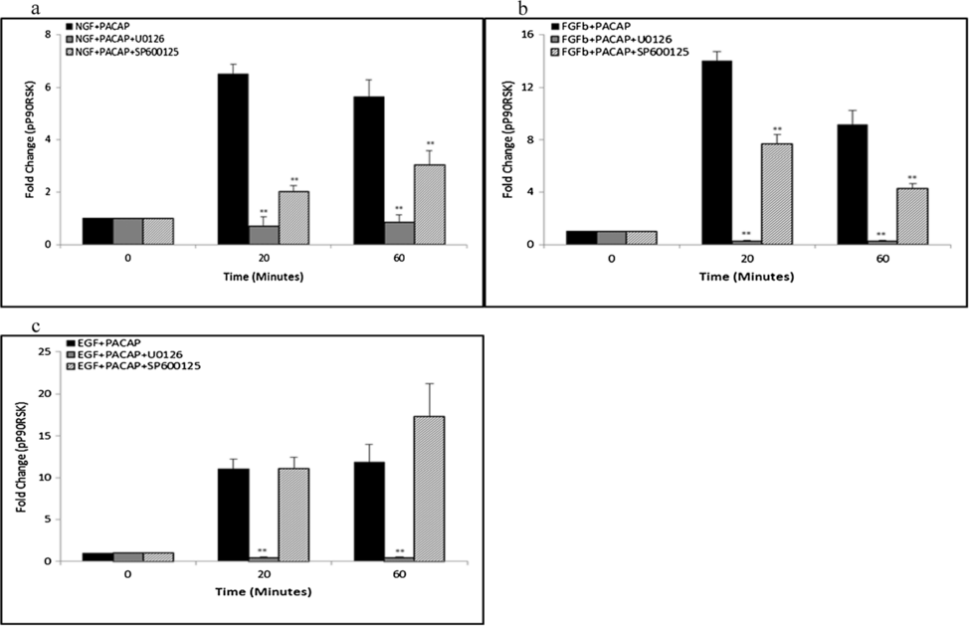
**P90RSK is regulated by Erk and JNK in the NP and FP systems, but only by Erk in the EP system. (a)**, **(b)**, **(c)** Time-course measurement of pP90RSK at 20 and 60 minutes following NGF (50 ng/ml)-PACAP (100 ng/ml), FGFb (50 ng/ml)-PACAP (100 ng/ml), and EGF (50 ng/ml)-PACAP (100 ng/ml) treatment, respectively, in the presence or absence of MEK inhibitor, U0126 (20 μM), or JNK inhibitor, SP600125 (10 μM). Significant differences between treatments with and without inhibitors were calculated using the paired Student’s *t*-test. A value of *p* < 0.05 was considered significant (**p < 0.01).

To validate the role of P90RSK as a downstream effector of synergistically activated JNK in the three systems, the phosphorylation level of P90RSK was examined after inhibition with SP600125. Surprisingly, treatment with SP600125 inhibited P90RSK phosphorylation in the NP (Figure 7a) and FP (Figure 7b), but not EP (Figure 7c), systems. These results strongly suggest that the regulation of P90RSK by the JNK pathway could be a critical determinant of JNK involvement in regulating synergistic neurite outgrowth.

In addition to JNK, P90RSK has also been reported to be a downstream target of Erk [37,38]. Unlike the case for JNK inhibition, inhibition of Erk activation with U0126 suppressed P90RSK phosphorylation in all three systems (Figure 7), providing further support for the role of P90RSK as an important mediator of neurite outgrowth. The total levels of Erk, JNK, and P90RSK were unchanged during the combinatorial growth factor-PACAP treatments both in the presence and absence of the inhibitors (Additional file 9: Figure S9).

## Discussion

**Figure 8:**
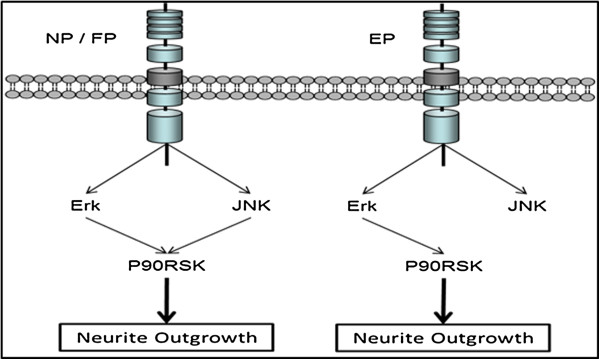
A schematic illustration of the different pathways used by the three different synergistic systems, NGF-PACAP (NP), FGFb-PACAP (FP), and EGF-PACAP (EP).

In this study, we demonstrated the involvement of distinct combinations of signaling pathways in mediating synergistic neurite outgrowth induced by PACAP and different growth factors (Figure 8). In these systems, Erk, JNK, and P90RSK were all found to be synergistically phosphorylated. However, synergistic JNK phosphorylation was not required for neurite outgrowth following stimulation with the combination of EGF and PACAP. Further investigations led to the crucial finding that the JNK-P90RSK link is critical to the involvement of JNK in regulating synergistic neurite outgrowth in some but not all growth factor-PACAP stimulation combinations.

cAMP-elevating agents have long been known to synergize with NGF [25,26], FGFb [24], and EGF [22,23] to enhance neurite outgrowth. Although the pathways used by these individual ligands to regulate neurite outgrowth have been widely studied, little is known about the mechanisms underlying synergistic neurite outgrowth. RSM-based analyses provide a means to quantitatively compare the degree of synergism between different treatments [29]. By such analyses, the degree of synergism in the EP system was found to be higher than those in the NP and FP systems, suggesting that different signaling pathways may regulate neurite outgrowth in these systems.

To determine the pathways involved in synergistic neurite outgrowth, four kinases were examined, each widely reported to be involved in PC12 cells differentiation: Erk [14,39,40], P38 [16,39], JNK [17,41], and Akt [18,40,42]. Interestingly, our results showed that Akt and P38 were activated following ligand stimulation but not involved in neurite outgrowth in all three systems. In agreement with this, inhibition of these two kinases also failed to suppress NGF-induced PC12 cells neurite outgrowth. These results were consistent with some of the earlier reports exploring neurite outgrowth [43-45] but not others [15-18,39-42]. A recent systems-based study revealed a two-dimensional Erk-Akt signaling code that was critical in governing PC12 cells proliferation and differentiation [46]. Thus, the controversy surrounding the involvement of P38 and Akt would be more adequately addressed using systems-based approaches in the future.

The sustained activation of Erk has been widely reported to be required for neurite outgrowth during differentiation [14,21,27,31]. Consistent with these reports, synergistic and sustained Erk phosphorylation was found to be involved in neurite outgrowth in all three growth factor-PACAP systems. This was especially evident in the EP system, where transient Erk activation was observed following treatment with EGF or PACAP alone. Similarly, synergistic and sustained JNK phosphorylation was observed in all three systems. Remarkably, inhibition of JNK led to reduced neurite outgrowth in the NP and FP systems, but enhanced outgrowth in the EP system. Although a previous study has found sustained JNK activation to be sufficient to induce PC12 cells differentiation [47], our results showed that sustained JNK activation in the EP system is insufficient to induce neurite outgrowth. These seemingly contradictory findings could imply that the kinetics of JNK activation alone is insufficient to determine if cells undergo differentiation. It is likely that JNK acts in conjunction with other signaling nodes to form a signaling network that regulates neurite outgrowth. Nonetheless, to the best of our knowledge, this is the first report demonstrating the involvement of JNK phosphorylation in synergistic neurite outgrowth.

We have shown that both Erk and JNK were synergistically phosphorylated in all three systems. This may occur through shared common upstream effectors [48] or through independent upstream effectors, such as PKA and Epac [49-51]. In preliminary experiments, we observed the involvement of PKA in neurite outgrowth in the EP but not NP system (data not shown); however, a complete understanding of the contribution of PKA and Epac in Erk and JNK activation remains to be determined.

Although synergistic JNK phosphorylation was observed in all three systems, it was not found to be involved in synergistic neurite outgrowth in the EP system. This suggests a possible difference in downstream signaling. P90RSK, which had previously been found to be required for PC12 cells differentiation [33], was also found to be synergistically activated in all three systems in our study. Interestingly, P90RSK was activated by JNK in the NP and FP, but not EP, systems. Although JNK-mediated activation of P90RSK has not been widely reported, it has been observed following ultraviolet exposure [34], insulin treatment [52], or transforming growth factor alpha treatment [53]. Consistent with previous findings [38], P90RSK was also regulated by Erk in our study. The co-regulation of targets by Erk and JNK is not uncommon, with previous studies showing that these two kinases regulate many common targets, including transcription factors [15,54,55], immediate early genes [56] and differentiation-specific genes [15,56,57]. Despite this, results from several studies have suggested that the binding sites of P90RSK for Erk and JNK are likely to be different [34,58], further indicating that P90RSK may be discretely regulated by the two kinases. Our finding of the differential regulation of P90RSK in the NP and EP systems in this study strongly suggests that these synergistic systems can serve as excellent models to decipher the mechanistic regulation of P90RSK by its upstream kinases, Erk and JNK. The contributions of Erk, JNK and P90RSK in the mechanism of axonal outgrowths of neurons *in vivo* and *in vitro* will require further clarification in future studies.

## Conclusions

In conclusion, our study has demonstrated distinct pathways involved in synergistic neurite outgrowth in different systems. Importantly, our findings of the underlying pathways involved in these systems have two key implications. First, some kinases such as JNK may be synergistically activated by multiple ligands but yet not necessarily involved in the synergistic neurite outgrowth process and that its involvement in neurite outgrowth is dependent on its interaction with P90RSK. Second, in the EP system, the increased synergy in neurite outgrowth and lack of JNK requirement for regulating the process suggest that PACAP synergizes differently with different growth factors to enhance neurite outgrowth. These findings reveal that synergistic of neurite outgrowths induced by multiple ligands involves the interplay of a network of signals.

## Abbreviations

NGF: Nerve growth factor; FGFb: Basic-fibroblast growth factor; EGF: Epidermal growth factor; PACAP: Pituitary adenylate cyclase-activating peptide; RSM: Response surface model; Erk: Extracellular signal-regulated kinase 1/2; JNK: c-Jun N-terminal kinase; P38: P38 mitogen-activated protein kinases; PI3K: Phosphatidylinositol 3-kinase; NP: NGF-PACAP; FP: FGFb-PACAP; EP: EGF-PACAP; cAMP: Cyclic adenosine monophosphate; PKA: Protein kinase A; Epac: Exchange protein activated by cAMP.

## Competing interests

The authors declare that they have no competing interest.

## Authors’ contributions

All authors contributed to the design of the experiments. KHS conducted all the experiments. KHS, LZ, and HPT contributed to the analyses of the data. KHS drafted the manuscript while LZ, GS, and HPT revised it critically. All authors read and approved the final manuscript.

## Supplementary Material

Additional file 1: Figure S1 Time-course profiles of activations of kinases upon PACAP, NGF, and NP treatments. Fold changes of **(a)** pErk, **(b)** pJNK, **(c)** pP38, and **(d)** pAkt from 0-1 hour. Click here for file

Additional file 2: Figure S2 Non-synergistic phosphorylation of P38 and Akt upon combinatorial NGF (0-50 ng/ml) and PACAP (0-100 ng/ml) treatments. **(a)** Time-course of P38 and Akt phosphorylations at 20 and 60 minutes following NGF-PACAP treatments. Phosphorylation levels of the proteins were analyzed by western blotting, and normalized to the levels of actin. Fold changes of **(b)** pP38, and **(c)** pAkt under (i) uni-ligand treatments, (ii) bi-ligand treatments at 10 ng/ml of NGF, and (iii) bi-ligand treatments at 50 ng/ml NGF. Significant differences between combinatorial experimental treatment of NGF-PACAP and summation of their individual effects were calculated using the paired Student’s *t*-test. A value of *p*<0.05 was considered significant. Click here for file

Additional file 3: Figure S3 Synergistic phosphorylation of Erk and JNK upon combinatorial NGF (0-50 ng/ml) and PACAP (0-100 ng/ml) treatments. Fold changes of **(a)** pErk, and **(b)** pJNK under (i) uni-ligand treatments, (ii) bi-ligand treatments at 10ng/ml of NGF, and (iii) bi-ligand treatments at 50 ng/ml NGF. Significant differences between combinatorial experimental treatment of NGF-PACAP and summation of their individual effects were calculated using the paired Student’s *t*-test. A value of *p*<0.05 was considered significant (**p<0.01). Click here for file

Additional file 4: Figure S4 Non-Synergistic phosphorylation of P38 and Akt upon FP and EP treatments. Time-course of quantified P38, and Akt phosphorylations at 20 and 60 minutes following **(a)** FGFb (50 ng/ml)-PACAP (100 ng/ml), and **(b)** EGF (50 ng/ml)-PACAP (100 ng/ml) treatment. Fold changes of (i) pP38, and (ii) pAkt were quantified by densitometry and normalized to the levels of actin. Significant differences between combinatorial experimental treatment of growth factor-PACAP and summation of their individual effects were calculated using the paired Student’s *t*-test. A value of *p*<0.05 was considered significant. Click here for file

Additional file 5: Figure S5 Total levels of Erk, JNK, P90RSK, Akt and P38 were not changed following treatments with ligands. The total protein levels were assayed at 20 and 60 minutes post-stimulation. The same control (C, at t=0 minutes) was used for both time-points. Click here for file

Additional file 6: Figure S6 Representative images of cells treated with growth factors-PACAP in the presence of inhibitors in the three systems. NP, FP and EP. Click here for file

Additional file 7: Figure S7 Positive controls for the kinase inhibitors following treatment with NGF (50 ng/ml). **(a)** Inhibition of Erk phosphorylation in the presence of U0126. **(b)** Inhibition of c-Jun phosphorylation in the presence of SP600125. **(c)** Inhibition of P38 phosphorylation in the presence of SB203580. **(d)** Inhibition of Akt phosphorylation in the presence of LY294002. Click here for file

Additional file 8: Figure S8 Synergistic phosphorylation of P90RSK upon combinatorial NGF (0-50 ng/ml) and PACAP (0-100 ng/ml) treatments. **(a)** Fold changes of pP90RSK under (i) uni-ligand treatments, (ii) bi-ligand treatments at 10 ng/ml of NGF, and (iii) bi-ligand treatments at 50 ng/ml NGF. (b) Representative images of cells treated with growth factors (50 ng/ml)-PACAP (100 ng/ml) in the presence of BRD7389 in the three systems. Significant differences between combinatorial experimental treatment of NGF-PACAP and summation of their individual effects were calculated using the paired Student’s *t*-test. A value of *p*<0.05 was considered significant (**p<0.01). Click here for file

Additional file 9: Figure S9 Total levels of Erk, JNK, and P90RSK were not changed following treatments with inhibitors. The total protein levels were assay at 20 minutes post-stimulation. A normalizer (NGF-PACAP co-treated cells) in each blot served as a control to normalize between different blots. U=U0126 (20 μM) and S=SP600125 (10 μM). Click here for file
