# Supplementary figures and images for: c-Jun N-terminal kinase in synergistic neurite outgrowth in PC12 cells mediated through P90RSK
Source: BMC Neurosci. 2013 Dec 12;14:153. doi: 10.1186/1471-2202-14-153 (PMC4029309; doi:10.1186/1471-2202-14-153)

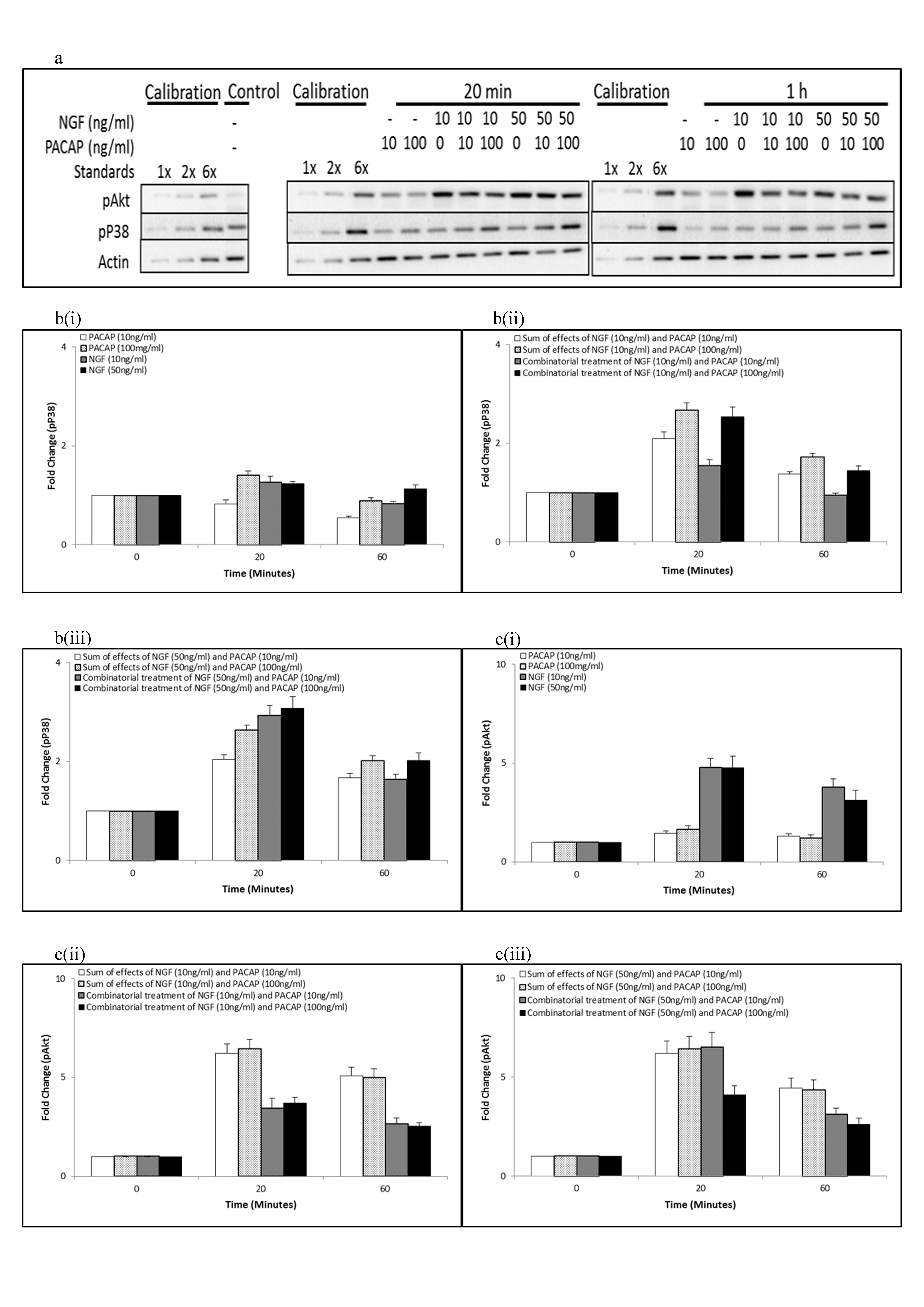

Supplement: Additional file 1: Figure S1 — Time-course profiles of activations of kinases upon PACAP, NGF, and NP treatments. Fold changes of (a) pErk, (b) pJNK, (c) pP38, and (d) pAkt from 0-1 hour. [file 1471-2202-14-153-S1.tiff]

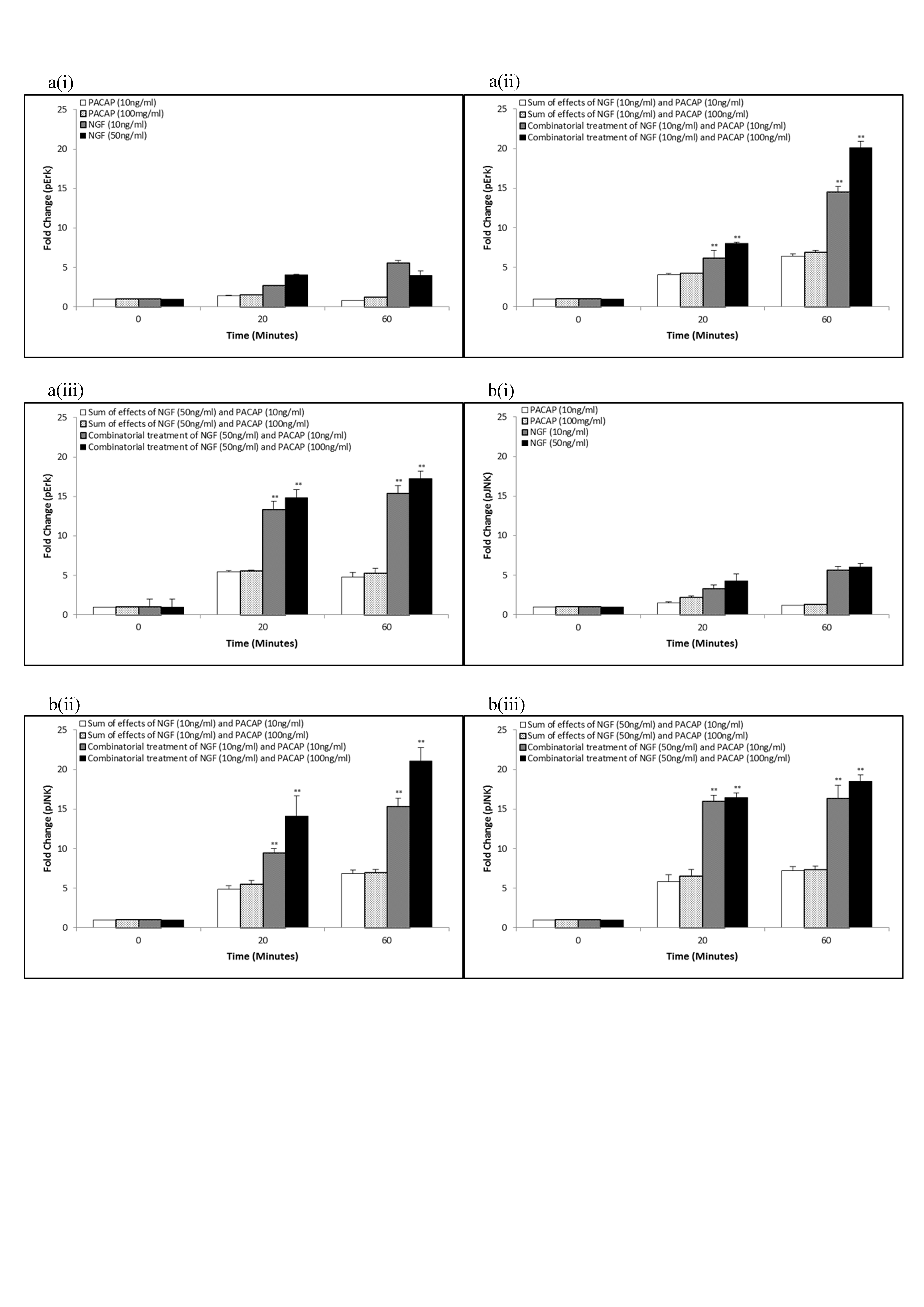

Supplement: Additional file 2: Figure S2 — Non-synergistic phosphorylation of P38 and Akt upon combinatorial NGF (0-50 ng/ml) and PACAP (0-100 ng/ml) treatments. (a) Time-course of P38 and Akt phosphorylations at 20 and 60 minutes following NGF-PACAP treatments. Phosphorylation levels of the proteins were analyzed by western blotting, and normalized to the levels of actin. Fold changes of (b) pP38, and (c) pAkt under (i) uni-ligand treatments, (ii) bi-ligand treatments at 10 ng/ml of NGF, and (iii) bi-ligand treatments at 50 ng/ml NGF. Significant differences between combinatorial experimental treatment of NGF-PACAP and summation of their individual effects were calculated using the paired Student’s t-test. A value of p<0.05 was considered significant. [file 1471-2202-14-153-S2.tiff]

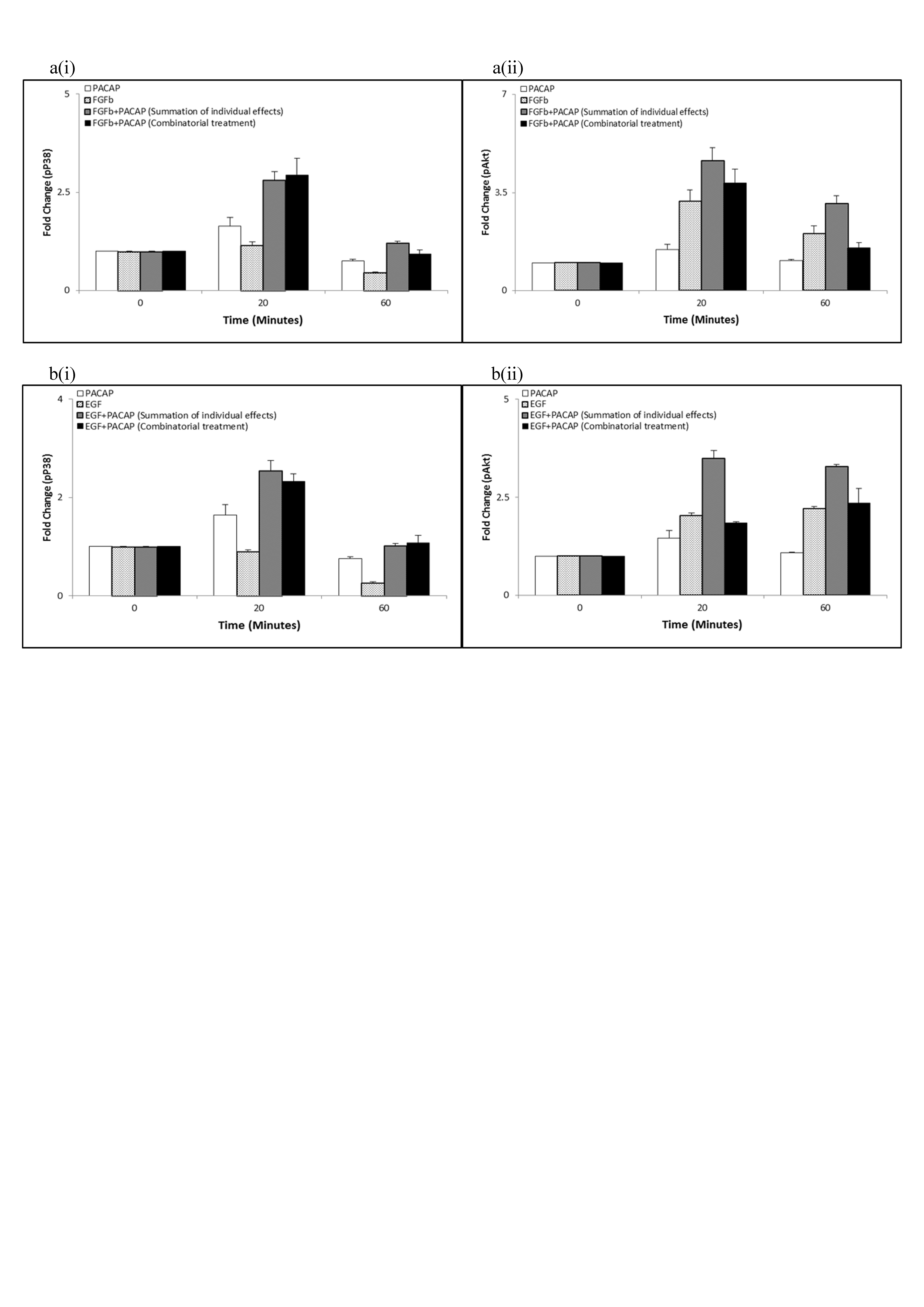

Supplement: Additional file 3: Figure S3 — Synergistic phosphorylation of Erk and JNK upon combinatorial NGF (0-50 ng/ml) and PACAP (0-100 ng/ml) treatments. Fold changes of (a) pErk, and (b) pJNK under (i) uni-ligand treatments, (ii) bi-ligand treatments at 10ng/ml of NGF, and (iii) bi-ligand treatments at 50 ng/ml NGF. Significant differences between combinatorial experimental treatment of NGF-PACAP and summation of their individual effects were calculated using the paired Student’s t-test. A value of p<0.05 was considered significant (**p<0.01). [file 1471-2202-14-153-S3.tiff]

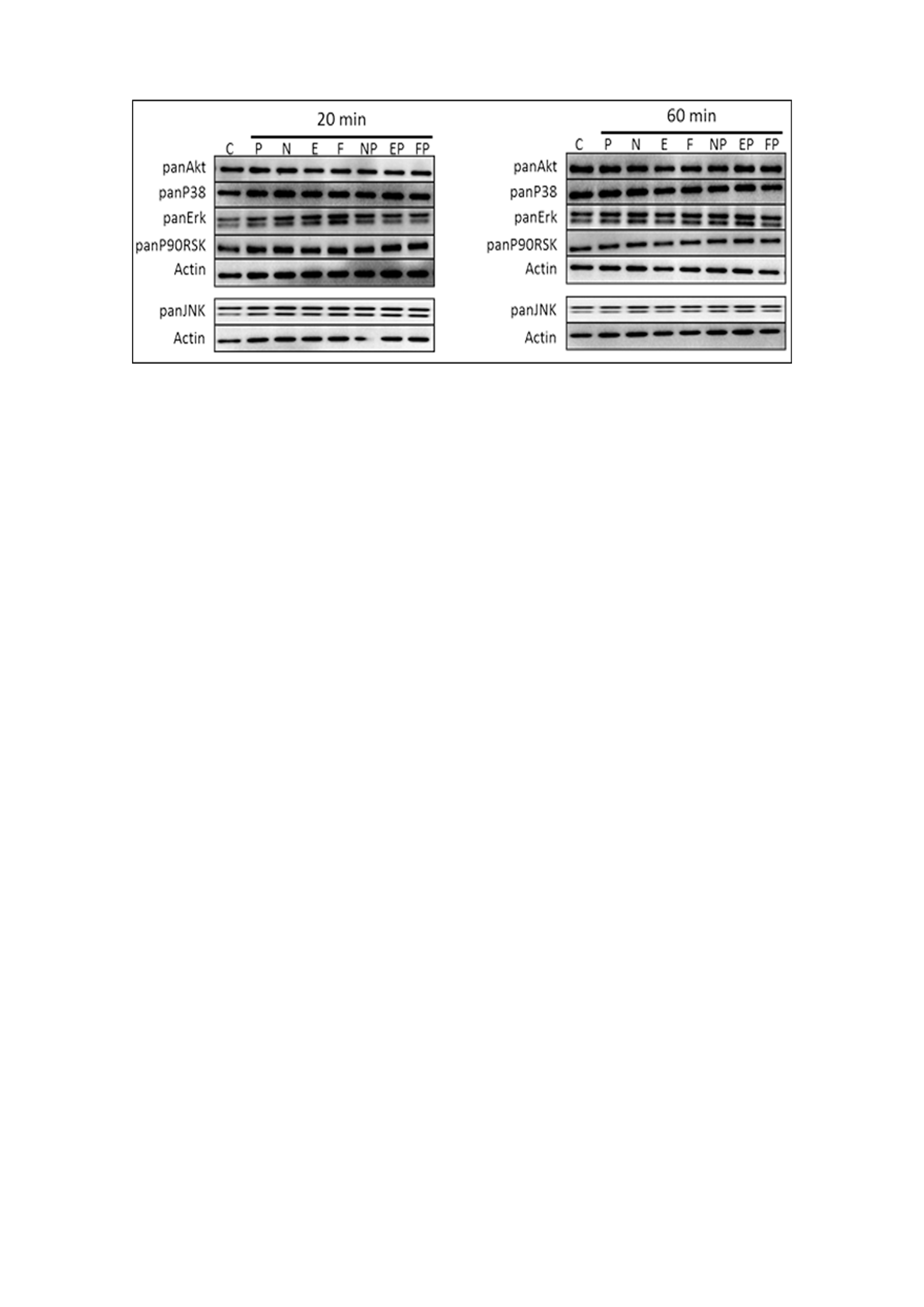

Supplement: Additional file 4: Figure S4 — Non-Synergistic phosphorylation of P38 and Akt upon FP and EP treatments. Time-course of quantified P38, and Akt phosphorylations at 20 and 60 minutes following (a) FGFb (50 ng/ml)-PACAP (100 ng/ml), and (b) EGF (50 ng/ml)-PACAP (100 ng/ml) treatment. Fold changes of (i) pP38, and (ii) pAkt were quantified by densitometry and normalized to the levels of actin. Significant differences between combinatorial experimental treatment of growth factor-PACAP and summation of their individual effects were calculated using the paired Student’s t-test. A value of p<0.05 was considered significant. [file 1471-2202-14-153-S4.tiff]

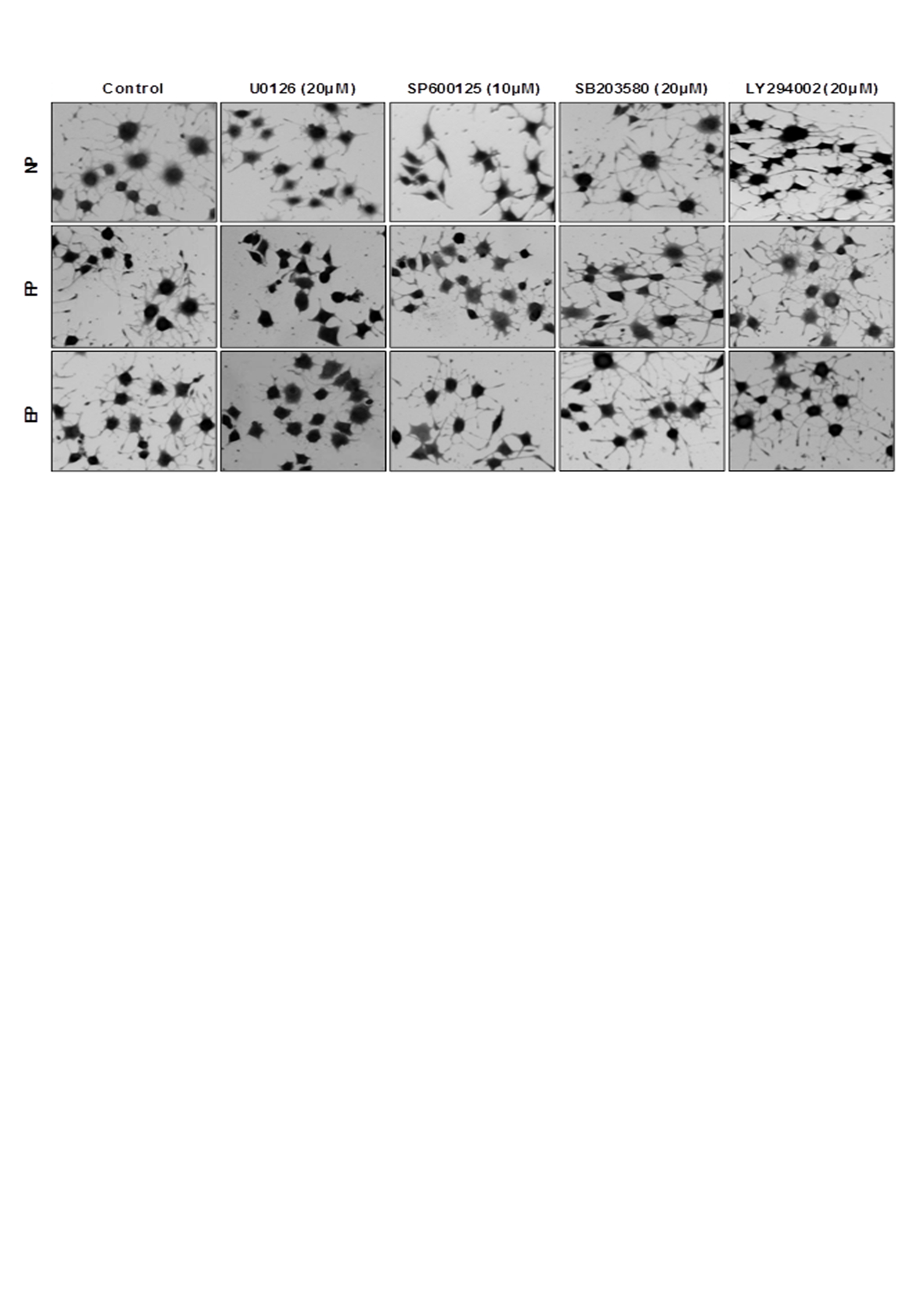

Supplement: Additional file 5: Figure S5 — Total levels of Erk, JNK, P90RSK, Akt and P38 were not changed following treatments with ligands. The total protein levels were assayed at 20 and 60 minutes post-stimulation. The same control (C, at t=0 minutes) was used for both time-points. [file 1471-2202-14-153-S5.tiff]

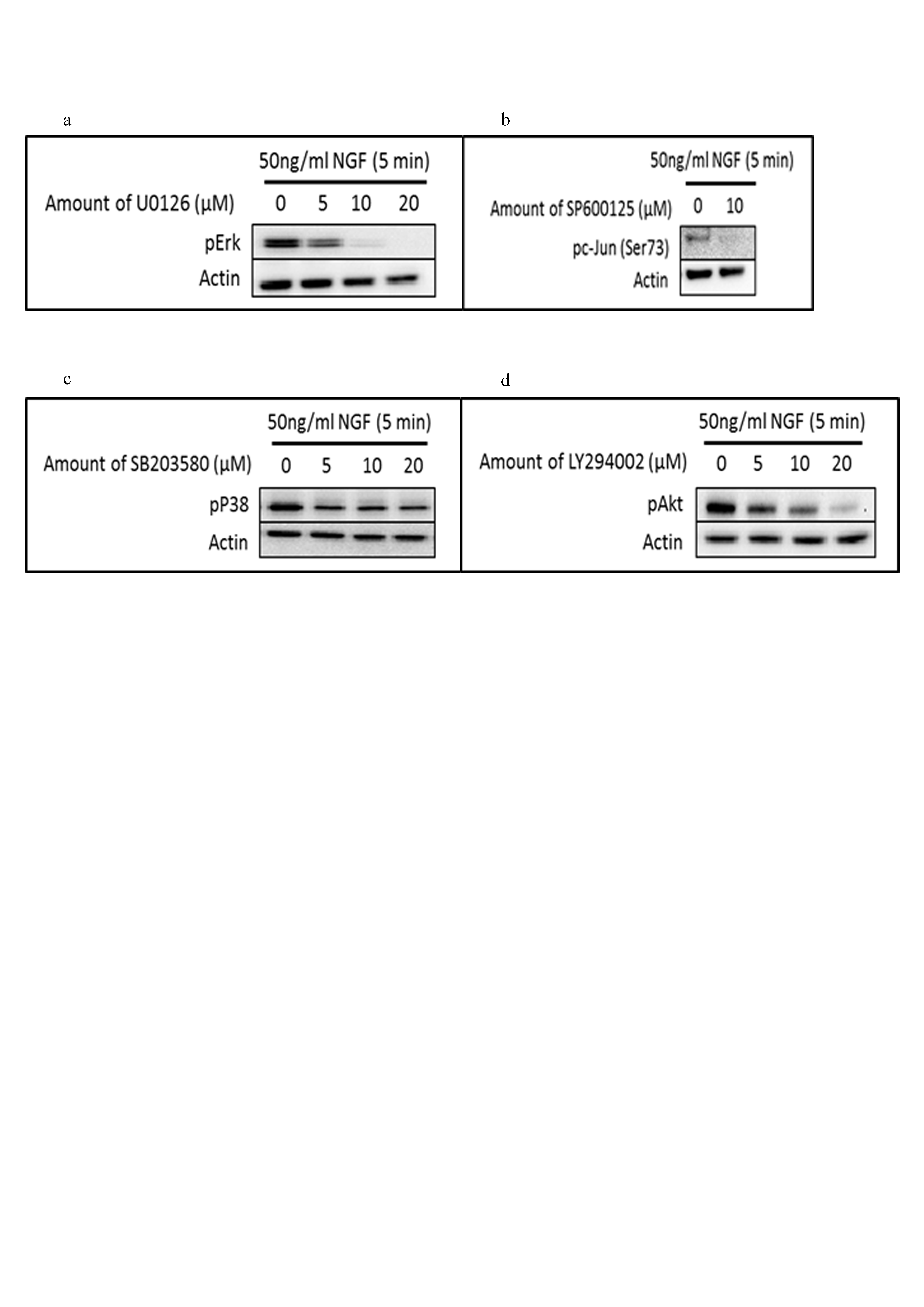

Supplement: Additional file 6: Figure S6 — Representative images of cells treated with growth factors-PACAP in the presence of inhibitors in the three systems. NP, FP and EP. [file 1471-2202-14-153-S6.tiff]

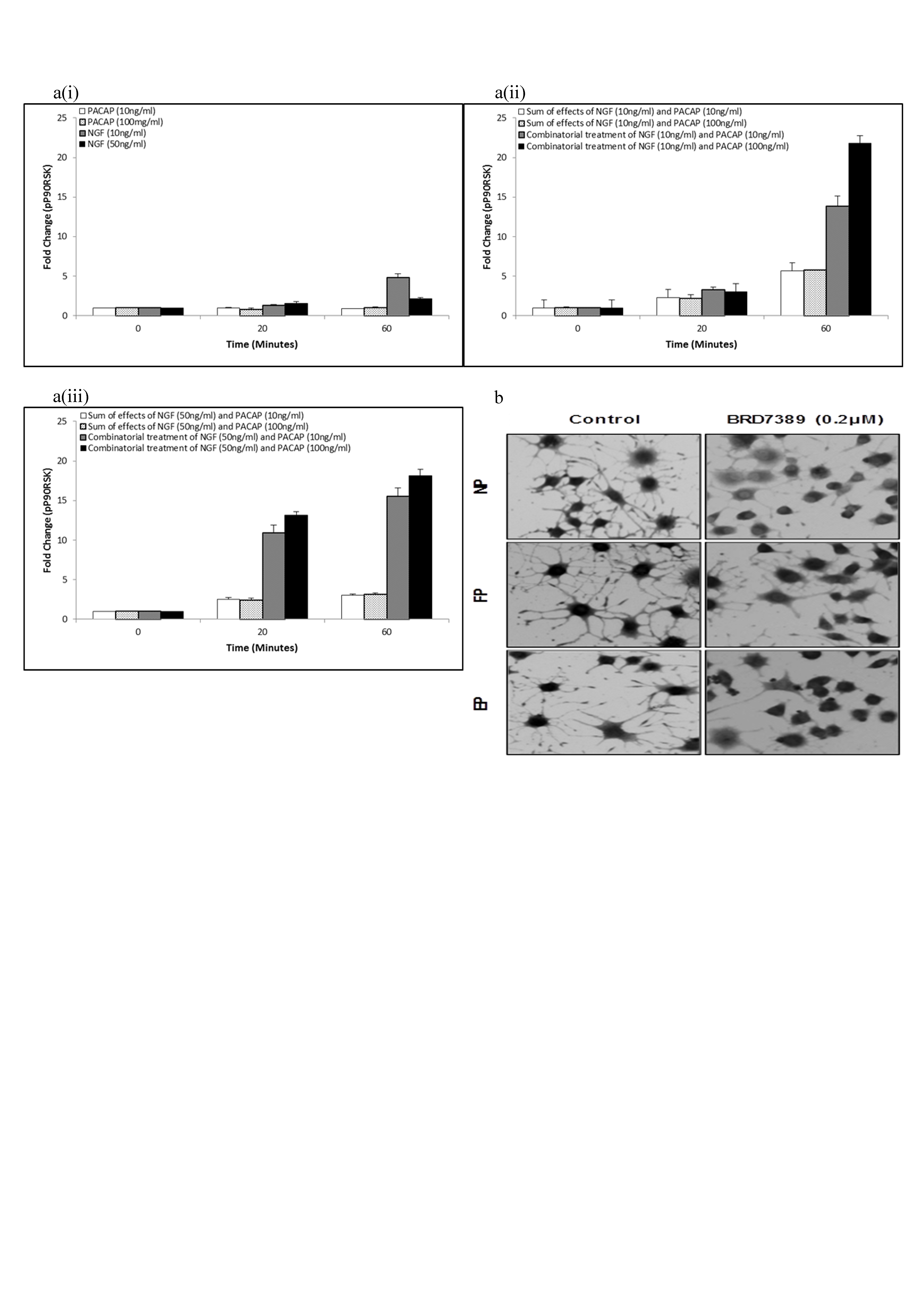

Supplement: Additional file 7: Figure S7 — Positive controls for the kinase inhibitors following treatment with NGF (50 ng/ml). (a) Inhibition of Erk phosphorylation in the presence of U0126. (b) Inhibition of c-Jun phosphorylation in the presence of SP600125. (c) Inhibition of P38 phosphorylation in the presence of SB203580. (d) Inhibition of Akt phosphorylation in the presence of LY294002. [file 1471-2202-14-153-S7.tiff]

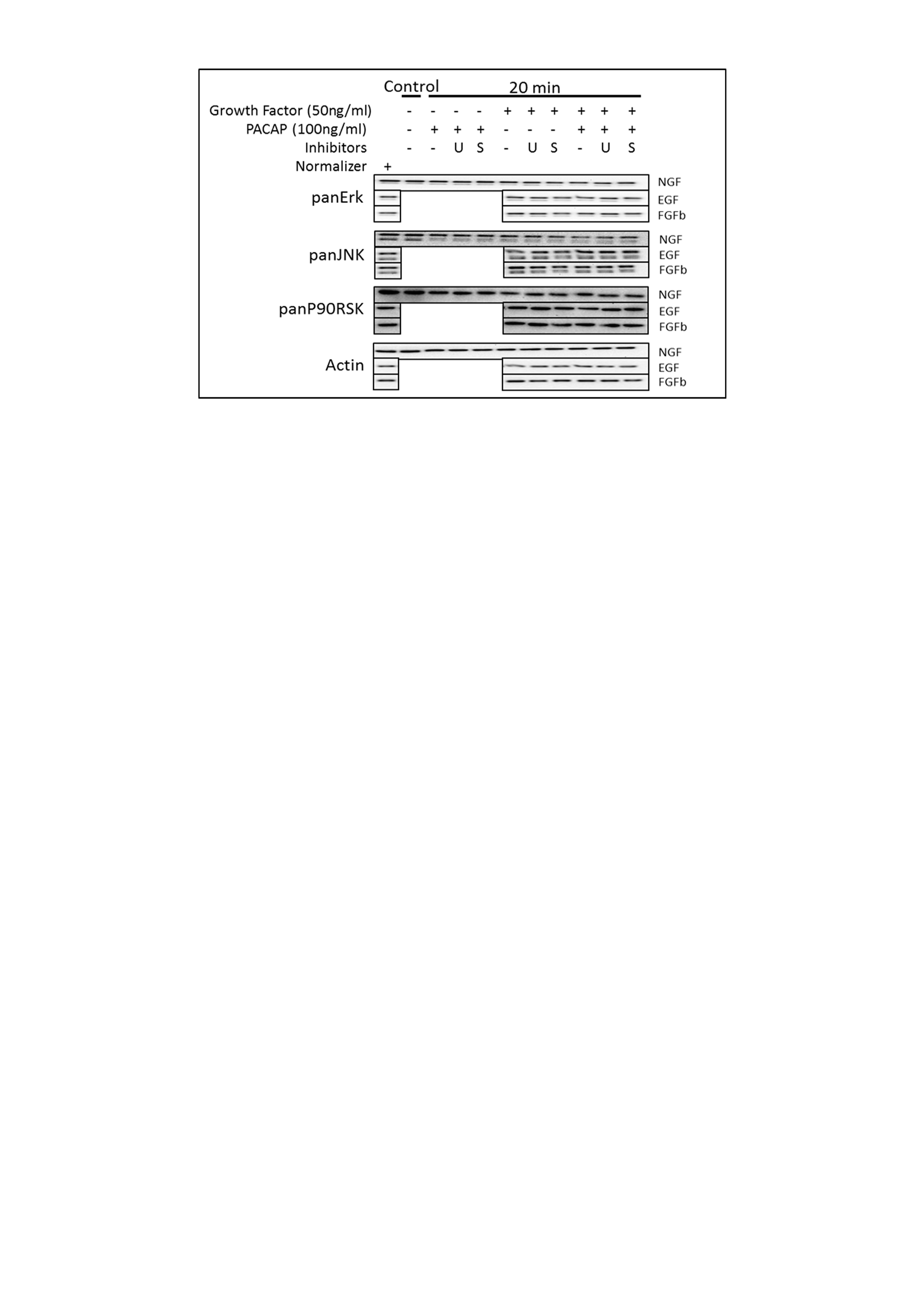

Supplement: Additional file 8: Figure S8 — Synergistic phosphorylation of P90RSK upon combinatorial NGF (0-50 ng/ml) and PACAP (0-100 ng/ml) treatments. (a) Fold changes of pP90RSK under (i) uni-ligand treatments, (ii) bi-ligand treatments at 10 ng/ml of NGF, and (iii) bi-ligand treatments at 50 ng/ml NGF. (b) Representative images of cells treated with growth factors (50 ng/ml)-PACAP (100 ng/ml) in the presence of BRD7389 in the three systems. Significant differences between combinatorial experimental treatment of NGF-PACAP and summation of their individual effects were calculated using the paired Student’s t-test. A value of p<0.05 was considered significant (**p<0.01). [file 1471-2202-14-153-S8.tiff]
